# Phenolic Compounds of Soybean Seeds from Two European Countries and Their Antioxidant Properties

**DOI:** 10.3390/molecules25092075

**Published:** 2020-04-29

**Authors:** Angelika Król-Grzymała, Ryszard Amarowicz

**Affiliations:** 1 Department of Biology and Biotechnology, Chair of Biochemistry, University of Warmia and Mazury, 10-748 Olsztyn, Poland; 2 Division of Food Science, Institute of Animal Reproduction and Food Research of the Polish Academy of Science, 10-748 Olsztyn, Poland; amaro@pan.olsztyn.pl

**Keywords:** soybean, seeds, phenolic compounds, isoflavones, antioxinants

## Abstract

There is only a small acreage of planted soybeans in northern Europe, as the global production of this crop is mainly dictated by the warmer temperatures needed for bountiful yields. The defense response of soybean plants to a cold climate entails the secretion of specific compounds which help mitigate oxidative stress, i.e., antioxidants, including phenolic compounds. The objective of this study was to examine differences in the concentrations of phenolic compounds, their antioxidant properties, and the concentration of key isoflavones (namely genistein, daidzein, malonyl daidzein, malonyl genistein, and daidzin) in the seeds of six soybean cultivars from two different regions of Europe, namely Poland and France. The total phenolic contents, isoflavone levels, and in vitro antioxidant capacities of soybean seeds from most of the investigated cultivars of northeast Europe were found to be greater than those from southwest Europe. The phenolic compounds of seed extracts are primarily responsible for the free-radical scavenging of soybeans. Factors regulating the production of phenolic compounds in the seeds have not been thoroughly elucidated. Hence, the results presented in this paper can be useful in the selection of soybean cultivars with higher levels of seed phenolics, because of their beneficial impact on human health and on the soybean’s defense mechanism against plant stresses.

## 1. Introduction

With respect to the carbon endoskeleton, phenolic compounds can be divided into four basic groups. One group consists of hydroxybenzoic acids (C6–C1), the second comprises phenylpropionic acids (C6–C3), the third is made up of flavonoids (including isoflavones) based on the carbon skeleton type C6–C3–C6, and the final group is phenolics with the carbon skeleton (C15)*_n_*, which forms tannins [1,2].

Isoflavones can be found in all plant parts, but their greatest concentration is in the seeds. Currently, 12 isoflavones have been identified in soybean seeds [3]. The most commonly reported soybean isoflavones in literature are two aglycones (daidzein, genistein), their glucosides (daidzin, genistein), and their malonylglysosides (malonyldaidzein, malonylgenistein) [4]. Since the 1990s, there has been an increasing interest in the bioactive properties of isoflavones. Many studies have shown that phenolic compounds, including isoflavones, bestow marked antioxidant properties, and that they are an important component of the plant’s non-enzymatic defense system towards abiotic and biotic stresses [5], e.g., thermal stress [6] or pathogens [3]. Furthermore, isoflavones have a positive effect on human health. Soybean isoflavones have effectively reduced lipid peroxidation in low density lipoprotein (LDL) cholesterol molecules, significantly reduced the risk of type II diabetes [7], and may also possess anti-cancer efficacy [8].

Under stress conditions, phenolic compounds are often synthesized in plants at elevated quantities. Studies have implied the induction of their synthesis by stress factors, which belong to biotic impacts, such as pathogens or lesions, and abiotic ones, like UV radiation, oxidative stress, water deficiency, and low temperatures [9,10,11,12]. Phenolic compounds can act as antioxidants, helping to support the endogenous enzymatic antioxidant systems in cells and to scavenge free radicals such as reactive oxygen species [13,14,15]. Different plant varieties often have varying tolerance to adverse environmental conditions. Phenolic compounds that are an important element of the plant’s defense system may have roles as biochemical markers of stress responses [16].

As an illustration, take into account the cold Polish climate and the important role of phenolic compounds in the protection of plants against various stresses. A possible solution is to plant cultivars that can better adapt to the unfavorable climate; in other words, which are less sensitive to chill stress. The purpose of this study was to compare the compositional profile and quantities of isoflavones found in soybean seeds from cultivars bred in Poland and France (the later grown in a much warmer climate) as well as reporting the antioxidant properties of the phenolic extracts so obtained from these seeds.

Common soybean (*Glycine max* L. Merr.) is an annual plant from Fabaceae (the bean family). Soybean seeds are a valuable source of nutrients: they contain roughly 40% protein, with their amino acid composition beneficial to human health, 20% cholesterol-free oil, including a high percentage of lipids rich in unsaturated fatty acids, and 33% carbohydrates as well as important macrominerals such as calcium and iron [17]. Soybean seeds also comprise functional components or bioactives, like oligosaccharides, phytosterols, anthocyanins, phytic acid, saponins, and isoflavones [18]. According to FOA statistics, 241 million tons of soybean seeds were produced worldwide in 2012. In northern Europe, however, soybeans are grown on a relatively small scale due to the cooler climate. The most favorable conditions for soybean cultivation in Europe are in the southeast, where the climate is temperate, and in the southwest, where the growing season for crops is longest [19].

It is common knowledge that interactions of plants with their environment will impact the biosynthesis of various secondary plant metabolites. Several years ago, phenolic compounds, an element of the secondary metabolism, were thought to be just metabolic by-products and practically worthless [20]. More recent studies have now demonstrated that phenolics play a significant signaling and protective role in plants. Furthermore, they can have a broad influence on the health of humans and animals eating these pulses [21].

## 2. Results

### 2.1. Total Phenolics Content (TPC)

The TPC results are shown in Table 1. The analysis revealed TPC values in seed extracts ranging from 10.3 to 13.7 mg/g of extract. Among the analyzed cultivars, cv. Mazowia possessed the highest content of phenolic compounds (13.07 mg/g of extract). Lower concentrations of phenolics were found in the cultivars Satina (12.5 mg/g of extract), Augusta (12.4 mg/g of extract), and Progres (12.04 mg/g of extract). The seed extract from Polish cv. Aldana contained even less phenolics (11.7 mg/g extract). Of all cultivars examined, the lowest TPC was determined in the French variety Isidor (10.3 mg/g of extract). In this work, the TPCs of all analyzed seeds were calculated per 100 g of fresh weight (FW) and dry weight (DW) of the seeds, as well as per seed (Table 1).

### 2.2. Scavening Activity and Reducing Power of Extracts

**Figure 1:**
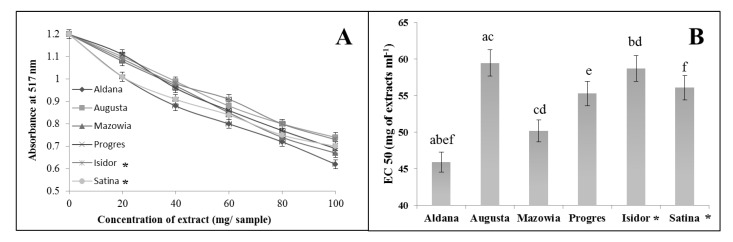
The capability to scavenge the free radical DPPH^•^ by extracts from soybean seeds (**A**). Half-maximal effective concentration (EC_50_) of extracts from soybean seeds to scavenge DPPH^•^ (**B**). Means with the same letters (a,b,c,d,e,f) are not significantly different (*p* < 0.05). Data represent the mean ± SD of four replicates. Varieties from France are marked with an asterisk (*), while the other varieties come from Poland.

The capability of prepared extracts from different soybean seed types to scavenge free radicals was determined by in vitro assays using two types of radicals: DPPH^•^ and ABTS^•+^. The extracts from all soybean cultivars demonstrated the capability to scavenge both of these free-radical species. The capability of extracts to scavenge DPPH^•^ is depicted in Figure 1A. Samples possessing the greatest antioxidant power were characterized by the lowest absorbance reading at 517 nm (Figure 1A). Considerable free-radical scavenging activity, at the highest extract concentration tested, was observed by extracts from seeds of the following varieties: Aldana (A_517_ – 0.62), Mazowia (A_517_ – 0.67), Progres (A_517_ – 0.68), Satina (A_517_ – 0.70), and Isidor (A_517_ – 0.73). The weakest free-radical scavenging capacity was identified in the extract from seeds of cv. Augusta (A_517_ – 0.74). The capability of soybean extracts to scavenge DPPH^•^ is expressed in half maximal effective concentrations (i.e., EC_50_ values), and the results are displayed in Figure 1B. Superior radical scavenging capacity is denoted by smaller EC_50_ values. The best EC_50_ value was observed in extracts from the variety Aldana (45.9 mg of extract/mL). EC_50_ values of other extracts were as follows: Mazowia (50.2 mg of extract/mL), Progres (55.3 mg of extract/mL), and the French variety Satina (56.1 mg of extract/mL). The weakest EC_50_ values were noted in extracts from seeds of the French variety Isidor (58.7 mg of extract/mL) and Polish variety Augusta (59.5 mg of extract/mL).

Seeds extracts for most of the analyzed cultivars exhibited a similar propensity to scavenge ABTS^•+^ (Table 2). Extracts prepared from seeds of the Polish cultivars Progres and Mazowia (50.6 and 50.0 µmol Trolox/g of extract, respectively) demonstrated the best capacity at quenching ABTS^•+^. Lower values were obtained for the following cultivars: Satina, Augusta, and Aldana (48.1, 46.2, and 45.4 µmol Trolox/g of extract, respectively). The lowest antioxidant capacity of seed extracts was determined for the French cultivar Isidor (44.1 µmol Trolox/g of extract). The reducing power of extracts obtained from seeds of different soybean cultivars was determined according to their capability to reduce trivalent iron ions (Fe^3+^) to divalent ones (Fe^2+^)—this was monitored via the ferric reducing antioxidant power (FRAP) assay. The results of these experiments are given in Table 2. Seed extracts from all analyzed soybean cultivars were able to reduce Fe^3+^. The greatest capacity to do so was observed in extracts from seeds of the cultivar Progres – 104.8 µmol Fe^2+^/g of extract. A weaker reducing power was determined in extracts from seeds of the following cultivars: Satina, Augusta, and Aldana (96.8, 94.7, and 92.2 µmol Fe^2+^/g of extract, respectively). Extracts from seeds of cv. Mazowia (91.2 µmol Fe^2+^/g of extract) and Isidor (90.3 µmol Fe^2+^/g of extract) exhibited the lowest reducing power of all soybean seed extracts tested.

### 2.3. Isoflavones Composition

**Figure 2:**
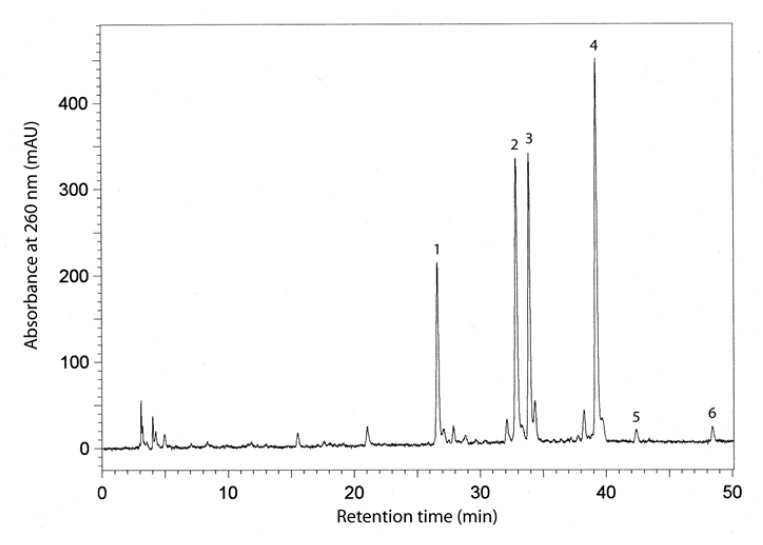
Chromatogram of resolved isoflavones in seed extracts of the soybean cultivar Aldana. The retention times of identified compounds were as follows: 1-daidzein-26 min; 2-genistein-33 min; 3-malonyldaidzein-34 min; 4-malonylgenistein-39 min; 5-daidzin-42 min; and 6-genistin-48 min.

Separation of the phenolic compounds in the seed extracts from the six soybean cultivars was achieved by reversed-phase high performance liquid chromatography (RP-HPLC). This procedure enabled us to identify the main phenolic compounds, which were found to be isoflavones. Figure 2 illustrates a typical chromatogram for the separation of the isoflavones in one of the soybean extracts, with retention times and absorbances for identified compounds. The following compounds were chromatographed and elucidated: daidzein, genistein, malonyldaidzein, malonylgenistein, daidzin, and genistin.

LC-QTOF analyses confirmed that malonyldaidzein and malonylgenistein were present in all analyzed extracts. The concentrations of isoflavones in seed extracts from the six investigated soybean cultivars are given in Table 3. Marked quantities of daidzein were detected in seed extracts from the cultivars: Mazowia, Progres, Satina, and Augusta (3.03, 2.66, 2.51, and 2.33 mg/g of extract, respectively), whereas significantly lower quantities of daidzein were determined in seed extracts from the cultivars: Aldana (2.44 mg/g of extract) and Isidor (1.73 mg/g extract). Considerable and similar amounts of genistein were determined in seed extracts from the cultivars: Progres, Mazowia, Augusta, and Satina (4.48, 4.3, 3.72, and 3.6 mg/g of extract), whereas markedly less of this isoflavone was detected in seed extracts from the soybean cultivars: Aldana (3.33 mg/g of extract) and Isidor (2.68 mg/g of extract). Another identified isoflavone derivative was malonyldaidzein: it occurred in large and similar concentrations in extracts from seeds of the following soybean cultivars: Mazowia, Aldana, Progres, Satina, Augusta (6.30, 5.90, 5.41, 5.23, and 5.15 mg/g of extract, respectively). On the other hand, less of this particular isoflavone was determined in the seed extract from cv. Isidor (3.83 mg/g of extract). The compound that appeared in the highest concentration in all analyzed samples was malonylgenistein. It occurred in similar concentrations in soybean seed extracts from the varieties: Augusta, Progres, Mazowia, Aldana and Satina (7.96, 7.28, 7.23, 7.04, and 6.52 mg/g of extract). Markedly less malonylgenistein was found in extracts from seeds of cv. Isidor (5.81 mg/g of extract). Another analyzed compound, daidzin, was detected in substantial concentrations in seed extracts from cv. Satina (0.3 mg/g of extract). In contrast, it was detected in markedly lower levels in extracts from seeds of the cultivars: Mazowia, Augusta, Progres, Aldana, and Isidor (0.21, 0.19, 0.19, 0.18, and 0.16 mg/g of extract). One final identifiable isoflavone was chromatographed, namely geistin. Its high and similar content was detected in extracts from seeds of the cultivars: Satina, Progres, and Mazowia (0.26, 0.25, 0.2 mg g^−1^ of extract, respectively). Markedly less genistin was found in soybean seed extracts from the cultivars Aldana, Augusta, and Isidor (0.18, 0.17 and 0.1 mg/g of extract). Finally, it should be mentioned that a high and comparable total content of isoflavones was characteristic for seed extracts from the cultivars Mazowia, Progres, Augusta, Aldana, and Isidor (21.3, 20.27, 19.52, 18.87, and 18.42 mg/g of extract). Conversely, a lower total content of isoflavones was determined in extracts from soybean seeds of cv. Isidor (14.31 mg/g of extract).

## 3. Discussion

Soybeans are grown mostly in the USA, Brazil, China, and India. This pattern of global distribution is mainly dictated by the high thermal and soil requirements of this plant [22,23]. Under northern Europe conditions, soybean seeds are often sown in chilled soil, and during spring, the emerging plants are often exposed to short spells of cold. This is the main cause for the plant’s uneven emergence, seed setting, delayed flowering, and often low yields [24]. The Polish register of crops, COBOR, contains eight soybean cultivars, namely Aldana, Augusta, Jutro, Mazowia, Nowiko, Progres, Pripyat, and Yanina. Of the varieties compared herein, it was only cv. Aldana that yielded above the average (104% of the model), whereas Augusta and Nowiko achieved 98% and 96% of the model yield. Regarding the other cultivars, there are no comparative data available to discuss their yields [25]. Kozak et al. [26] verified that the main determinants of seed yield are the minimum air temperature, amplitude of temperatures, and mean daily air temperature.

The main purpose of this study was to compare the quantities and composition of phenolic compounds in seeds of Polish and foreign (i.e., French) soybean cultivars. It is rather uncertain what factors regulate the production and distribution of phenolic compounds in soybean seeds. It is believed that an elevated level of phenolics in seeds protects them better from the harmful impact of both biotic and abiotic stresses. It is also claimed that these compounds can bestow beneficial effects on the human health. The present study found that the content of phenolic compounds in soybean seeds from most of the Polish cultivars analyzed (Aldana, Mazowia, and Progres) was higher than those in the French varieties (Isidor and Satina), thereby possibly indicating an important role of phenolic compounds in the adaptation of Polish cultivars to the local climatic conditions. Seeds of the Polish cultivar Augusta possessed a much lower content of phenolic compounds than any of the other Polish varieties. In fact, similar to the levels detected in the French cultivars. Furthermore, it was noted that this cultivar was characterized by a low yield based on the climatic conditions of Poland [25]. Phenolic extracts from these seeds were also distinguished by a low free-radical scavenging capacity.

Prakash et al. [27] analyzed 30 soybean cultivars from India, of which the highest content of phenolic compounds was determined for cv. Kalitur, whose seeds are black. In another study, it was shown that the phenolic compounds were partitioned differently between the top and bottom seed nodes [28]. Another finding indicated that the average TPC in green soybean sprouts was higher than in yellow counterparts when grown under dark conditions [29]. Other researchers have noted that phenolic metabolism in germinating soybean seeds is much more intensive in response to low temperature and osmotic stress, and remains at a high level during recovery after this stress [30]. Under stress conditions, the amount and composition of identified phenolic acids and isoflavones changes. This indicates the important role of phenolic compounds in alleviating the effects of abiotic stress during the germination of soybean seeds, and offers new perspectives for further investigation. Of particular note are phytoalexins, which are synthesized intensively when a plant is invaded by fungi following a bacterial or viral infection. Pisatin is the most thoroughly investigated phenolic phytoalexin. It is an isoflavonoid synthesized in pea pods after an infection with brown-rot fungus. The latest proteomic research has shown that 6a-hydroxymaackian-3-*O*-methyltransferase, which is involved in the synthesis of pisatin, was present, but only under osmotic stress conditions, thereby denoting its key role in the acquisition of stress tolerance by plants [31]. The identified protein, 6a-hydroxymaackian methyltransferase, can serve as an object for an engineering strategy, involved in certain new plant varieties that will be more resistant to unfavorable environmental conditions.

Isoflavones, a class of flavonoid in soybean seeds, have been found to possess important secondary compounds with many chemical actions. Especially useful seems to be their antioxidant and anticancer actions [29]. Numerous studies have demonstrated a correlation between isoflavone contents and the soybeans cultivation environment [28,32,33].

The soybean phenolic extracts analyzed in this study by RP-HPLC contained the following six isoflavonoids: daidzein, genistein, daidzin, genistin, malonyldadzein, and malonylgenistein. These are the major soybean flavonoids, as has been reported by numerous researchers [4,33,34,35,36,37]. These isoflavonoids were detected in high concentrations in the seed extracts, and ranged from 14.31 to 21.3 mg/g of extract. This study indicates that, despite differences in quantities of individual compounds in the various seed extracts, only extracts from the seeds of the French cultivar Isidor were characterized by a significantly lower total content of isoflavones than any others. The total content of isoflavones in seed extracts from all analyzed cultivars here is within the range of the levels determined for seeds of different soybean cultivars [13,32,38,39]. According to Seo and Morr [40], the content of isoflavonoids constitutes about 72% of all phenolic compounds in soybean seeds. Isoflavones are very powerful antioxidants, especially those occurring in their free form, such as genistein and dadzein, which possess the highest antioxidant power of all isoflavones in foodstuffs [33]. Depending on the quantity and composition of isoflavones, different types of soybean and soybean products demonstrate varied antioxidant activity [41].

The research findings reported in this paper show that seed extracts from the analyzed soybean varieties have a high capacity to reduce the DPPH radical and the ABTS radical cation. The weakest power to scavenge free radicals was observed for cv. Augusta, while the other cultivars, namely Aldana, Mazowia, Progres, Isidor, and Satina, demonstrated a similar or greater scavenging power. Seed phenolic extracts from the aforementioned cultivars from India are highly varied in their capacity to scavenge free radicals. Analogous to our investigation, a correlation was determined between the content of phenolic compounds and their capability to scavenge free radicals by extracts prepared from these seeds [26]. Malencic et al. [42] also demonstrated a linear dependence between the content of phenolic compounds in seeds and the capacity of extracts isolated from these seeds to scavenge free radicals.

## 4. Materials and Methods

### 4.1. Chemicals

Folin-Ciocalteu’s phenol reagent (FCR), (+)-catechin, sodium carbonate, gallic acid, 2,2′-diphenyl-1-picrylhydrazyl (DPPH), 2,2′-azinobis-(3-ethylbenzothiazoline-6-sulfonic acid) (ABTS), 2,4,6-tri(2-pyridyl)-*S*-triazine (TPTZ), 6-hydroxy-2,5,7,8-tetramethyl-chroman-2-carboxylic acid (Trolox), trifluoroaceitc acid, daidzein, genistein, malonyldaidzein, malonylgenistein, daidzin, and genistin were obtained from the Sigma-Aldrich Chemical Company (St. Louis, MO, USA). Methanol, acetone, hexane, acetonitrile, acetic acid, ferric chloride, and potassium persulfate were acquired from Chempur (Piekary Śląskie, Poland).

### 4.2. Plant Material

The biological material used in these experiments comprised seeds of six cultivars of common soybeans (*Glycine max* L.), four Polish cultivars, namely Aldana, Augusta, Mazowia, and Progres, and two French ones, namely Isidor and Satina. The Polish cultivars originated from the Central Research Station on Crop Cultivars in Słupia Wielka, while the French ones were supplied by Actisem (le Jardin, Francescas, France). The dry matter of seeds was determined by drying at 105 °C for 24 h.

### 4.3. Phenolic Compounds Extraction

Seeds from six varieties of soybeans were ground in a coffee mill and defatted with hexanes using a Soxhlet apparatus for 6–8 h. Phenolic compounds were extracted from the defatted raw seeds with 80% (*v*/*v*) acetone and 80% (*v*/*v*) methanol at a solids-to-solvent ratio of 1:10 (*w*/*v*) at 50 °C for 30 min [43]. The extraction was carried out in glass bottles in a shaking water bath (Elpan 357, Wrocław, Poland). The extraction was repeated two more times, the supernatants were filtered and combined. The organic solvent was then evaporated under vacuum using a Büchi rotary evaporator (Flawil, Switzerland) and water bath set at 40 °C. The resultant aqueous solutions was frozen and lyophilized.

### 4.4. Total Phenolics Content (TPC)

The TPC in extracts was determined using Folin–Ciocalteu’s phenol reagent (FCR), Sigma-Aldrich Chemical Company (St. Louis, MO, USA) [44]. To 0.25 mL of a methanolic solution of phenolic extract (containing between 0.1 to 0.2 mg of extract) was added 0.25 mL of a saturated sodium carbonate solution, 0.25 mL Folin–Ciocalteu’s phenol reagent, and 4 mL of deionized water. The mixture was vortexed and then incubated at room temperature in dark for 0.5 h. After this period, the absorbance was read at 725 nm using a spectrophotometer. (+)-Catechin was employed as the standard.

### 4.5. Scavenging of the DPPH Radical

The scavenging effect of phenolic compounds from the extracts was measured accordance to Yen and Chen [45]. Briefly, 0.1 mL of a methanolic solution of phenolic extract (containing 0.05 to 0.1 mg of extract) was mixed with 2 mL of deionized water, to which a freshly prepared methanolic solution of DPPH^•^ (1 mM, 0.25 mL), Sigma-Aldrich Chemical Company (St. Louis, MO, USA) was then added. The mixture was vortexed and incubated at room temperature for 20 min. The absorbance was read at 517 nm using a spectrophotometer. The results were presented as half maximal effective concentrations to quench the DPPH^•^ (EC_50_) in units of mg of extract/mL.

### 4.6. Trolox Equivalent Antioxidant Capacity (TEAC)

The TEAC assay was carried out in accordance with Huang et al. [46]. Briefly, 0.1 mL of a methanolic solution of phenolic extract (containing 0.1 to 0.5 mg of extract) was added to 0.1 mL of methanol (Chempur (Piekary Śląskie, Poland)) and 2 mL of ABTS^•+^ (Sigma-Aldrich Chemical Company (St. Louis, MO, USA)). The mixture was vortexed and incubated in the dark at 32 °C for 20 min. The absorbance was read at 734 nm using a spectrophotometer. A calibration curve was prepared using Trolox (Sigma-Aldrich Chemical Company (St. Louis, MO, USA)) as the standard. Results were reported as µmol Trolox equivalents/g of extract.

### 4.7. Ferric-Reducing Antioxidant Power

Reducing powers of the phenolic extracts were determined using the FRAP assay [47]. Briefly, 0.1 to 0.5 mg of phenolic extract was dissolved in 0.1 mL of deionized water, to which 3 mL of freshly prepared FRAP reagent (acetic acid buffer of pH 3.6 TPTZ (2,4,6-tris(2-pyridyl)-*S*-triazine) was added. The mixture was incubated for 30 min at ambient temperature. After incubation, the absorbance was read at 593 nm. The results were expressed as µmol Fe^2+^/g of extract.

### 4.8. Isoflavones Analysis

An RP-HPLC Shimadzu system (Shimadzu, Kyoto, Japan) was used to analyze the isoflavones in the extracts from the soybean seeds. The phenolic extracts were dissolved in 80% (*v*/*v*) methanol and passed through a 0.45 μm nylon filter prior to injection [48]. The separation was carried out using a gradient elution. The A solvent consisted of a mixture of water, acetonitrile, and trifluoroaciteic acid (95:5:1 *v*:*v*:*v*), while the B solvent comprised acetonitrile and trifluoroaceitc acid (Chempur (Piekary Śląskie, Poland, 100:1 *v*:*v*)). The flow of solvent B in the linear gradient was from 0% to 40% over 60 min. The analysis of each phenolic extract was performed in triplicate. Linearity of the detector responses was determined for daidzin and genistein (range of 0.05 to 0.15 mg/mL), daidzein (range of 0.01 to 0.04 mg/mL), and genistin (range of 0.02 to 0.08 mg/mL). The relation between area under a peak and standard concentrations was characterized by a correlation coefficient of 0.997 for genistein, and 0.998 for daidzin, daidzein, and genistein.

Next, the analysis of soybean isoflavones were continued using a micro-HPLC system (LC200, Eksigent, Framingham, MA, USA) consisting of a dual-channel pump, column oven, autosampler, and system controller. The micro-HPLC was coupled to a 5600 QTOF mass spectrometer (SCIEX, Ontario, Canada). Five µL of each sample were injected onto the chromatography column, HALO C18, 2.7 µm, 0.5 × 50 mm (Eksigent, Framingham, MA, USA). All chromatographic determinations were performed at 45 °C, at a mobile phase flow rate of 15 µL/min, using the following gradient: 5–5–90–90–5–5% B in 0–0.5–1.8–2.8–3–5 min (phase A: water/formic acid, 99.1/0.9 (*v*/*v*) phase B: acetonitrile/formic acid, 99.1/0.9 (*v*/*v*)).

QTOF conditions in the negative-ion mode were as follows: nitrogen curtain gas, 25 L/min; ion-spray source voltage, −4500 V NEG; temperature, 350 °C; nebulizer gas 1, 35 L/min; turbo gas, 30 l/min; Q1/Q2, DP −90/−80 V; CE −10/−10 V (CE10/30); and CES 15 V.

QTOF conditions in the positive-ion mode were as follows: nitrogen curtain gas, 25 L/min; ion-spray source voltage, 5500V POS; temperature, 350 °C; nebulizer gas 1, 35 L/min; turbo gas, 30 L/min; Q1/Q2, DP 90/80 V; CE 10/10 V (CE10/30); and CES 15 V.

### 4.9. Statistical Analysis

Antioxidant assays and HPLC separations and identification of isoflavones were performed for at least three repetitions. All results were presented as means ± standard deviations (SD). Significance of differences among mean values were analyzed using a nonparametric test (Friedman and Wilcoxon test) at a level of *p* < 0.05 in software Statistica.

## 5. Conclusions

The research presented in this paper has demonstrated that the total content of phenolic compounds and antioxidant capacities in extracts of soybean seeds for most of the examined Polish cultivars was higher than those of the French varieties. Thus, it can be concluded that it is the phenolic compounds enclosed in seed extracts that are mostly responsible for the free-radical scavenging capacity in soybean seeds. Isoflavones were determined in all investigated soybean extracts: genistein, daidzein, malonyl daidzein, malonyl genistein, and daidzin were detected. Noteworthy is that the factors regulating the production of phenolic compounds in seeds have not been thoroughly recognized in literature. Consequently, many teams of researchers conduct experiments in order to select soybean seeds with higher levels of seed phenolics, because of their beneficial impact on human health and on soybean’s defense mechanism against both biotic and abiotic stresses.

## Figures and Tables

**Table 1:** The total phenolics contents (TPCs) in soybean seeds.

Variety	mg/g Extract	mg/100g FW	mg/100g DW
Aldana	11.7 ± 0.2 ^c^	2.13 ± 0.04 ^b,c^	2.32 ± 0.03 ^b^
Augusta	12.4 ± 0.1 ^b^	2.13 ± 0.02 ^b^	2.26 ± 0.02 ^c^
Mazowia	13.7 ± 0.1 ^a^	2.48 ± 0.02 ^a^	3.01 ± 0.03 ^a^
Progres	12.04 ± 0.2 ^c^	2.07 ± 0.02 ^c^	2.34 ± 0.02 ^b^
Isidor *	10.3 ± 0.3 ^d^	1.71 ± 0.05 ^d^	1.77 ± 0.02 ^e^
Satina *	12.5 ± 0.1 ^b^	2.06 ± 0.02 ^c^	2.16 ± 0.02 ^d^

Means with the different letters (a,b,c,d,e) in the column are significantly different (*p* < 0.05). Data represent the mean ± SD of four replicates Varieties from France are marked with an asterisk (*), the other varieties come from Poland.

**Table 2:** Trolox equivalent antioxidant capacity (TEAC) and ferric-reducing antioxidant power (FRAP) in soybean seed extracts.

Variety	TEAC	FRAP
	µmol Trolox/g of Extract	µmol Fe^2+^/g of Extract
Aldana	45.4 ± 0.3 ^c,d,e^	92.2 ± 6.7 ^a,b^
Augusta	46.2 ± 1.1 ^d^	94.7 ± 6.4 ^a,b^
Mazowia	50.0 ± 0.3 ^bc^	91.2 ± 4.1 ^b^
Progres	50.6 ± 0.2 ^a^	104.8 ± 4.8 ^a^
Isidor *	44.1 ± 0.4 ^e,d^	90.3 ± 5.6 ^b^
Satina *	48.1 ± 1.4 ^c^	96.8 ± 4.8 ^a^

Means with the different letters (a,b,c,d,e) in the column are significantly different (*p* < 0.05). Data represent the mean ± SD of four replicates. Varieties from France are marked with an asterisk (*), the other varieties come from Poland.

**Table 3:** Content of isoflavones in extracts from soybean seeds.

Variety	Isoflavones (mg/g^-^of Extract)						
	Daidzein	Genistein	Malonyldaidzein	Malonylgenistein	Daidzin	Genistin	Total
Aldana	2.24 ± 0.13 ^d^	3.33 ± 0.19 ^c^	5.91 ±0.34 ^a,b^	7.04 ± 0.44 ^a^	0.18 ± 0.03 ^b^	0.18 ± 0.02 ^c^	18.87 ± 2.23 ^a,b^
Augusta	2.33 ± 0.22 ^c,d^	3.72 ± 0.21 ^b,c^	5.15 ± 0.21 ^c^	7.96 ± 0.69 ^a^	0.19 ± 0.01 ^b^	0.17 ± 0.03 ^c^	19.52 ± 2.17 ^a,b^
Mazowia	3.05 ± 0.16 ^a^	4.31 ± 0.31 ^a,b^	6.33 ± 0.34 ^a^	7.23 ± 0.89 ^b^	0.21 ± 0.02 ^b^	0.21 ± 0.01 ^b,c^	21.3 ± 1.74 ^a^
Progres	2.66 ± 0.14 ^b^	4.48 ± 0.33 ^a^	5.41 ± 0.53 ^c^	7.28 ± 1.03 ^b^	0.19 ± 0.02 ^b,c^	0.25 ± 0.03 ^b^	20.27 ± 1.59 ^a,b^
Isidor *	1.73 ± 0.05 ^e^	2.68 ± 0.14 ^d^	3.83 ± 0.39 ^d^	5.81 ± 0.12 ^b^	0.16 ± 0.01 ^c^	0.11 ± 0.01 ^d^	14.31 ± 1.14 ^c^
Satina *	2.51 ± 0.06 ^b,c^	3.62 ± 0.19 ^b,c^	5.23 ± 0.31 ^b,c^	6.52 ± 0.62 ^b^	0.30 ± 0.03 ^a^	0.26 ± 0.01 ^a^	18.42 ± 1.33 ^b^

Means with different letters (a,b,c,d,e) in the column are significantly different (*p* < 0.05). Data represent the mean ± SD of three replicates. Varieties from France are marked with an asterisk (*), the other varieties come from Poland.
